# Regulatory mechanisms of TGF‐β1‐induced fibrogenesis of human alveolar epithelial cells

**DOI:** 10.1111/jcmm.12918

**Published:** 2016-07-15

**Authors:** Lin Shi, Nian Dong, Xiaocong Fang, Xiangdong Wang

**Affiliations:** ^1^ Zhongshan HospitalShanghai Institute of Clinical BioinformaticsFudan University Center for Clinical BioinformaticsZhongshan Hospital Institute of Clinical Science of Fudan UniversityShanghaiChina

**Keywords:** TGF‐β1, PI3K, CTGF, EMT, fibrosis

## Abstract

Pulmonary fibrosis is characterized by an extensive activation of fibrogenic cells and deposition of extracellular matrix (ECM). Transforming growth factor (TGF)‐β1 plays a pivotal role in the pathogenesis of pulmonary fibrosis, probably through the epithelial‐ to‐mesenchymal transition (EMT) and ECM production. The present study investigates potential mechanism by which TGF‐β1 induces EMT and ECM production in the fibrogenesis of human lung epithelial cells during pulmonary fibrosis. The expression of EMT phenotype and other proteins relevant to fibrogenesis were measured and the cell bio‐behaviours were assessed using Cell‐IQ Alive Image Monitoring System. We found that TGF‐β1‐induced EMT was accompanied with increased collagen I deposition, which may be involved in the regulation of connective tissue growth factor (CTGF) and phosphoinositide 3‐kinase (PI3K) signalling pathway. Treatment with PI3K inhibitors significantly attenuated the TGF‐β1‐ induced EMT, CTGF expression and collagen I synthesis in lung epithelial cells. The interference of CTGF expression impaired the basal and TGF‐β1‐stimulated collagen I deposition, but did not affect the process of EMT. Our data indicate that the signal pathway of TGF‐β1/PI3K/CTGF plays an important role in the fibrogenesis of human lung epithelial cells, which may be a novel therapeutic approach to prevent and treat pulmonary fibrosis.

## Introduction

Pulmonary fibrosis is one of chronic lung diseases, with a rapid progression, high mortality and poor response to therapy. Myofibroblast was regarded as a driver during the process of fibrogenesis 1, while lung epithelial cell may act as receptor and initiator for the secondary process to form the fibrogenesis 2, 3. The previous study demonstrated that up to 30% of myofibroblast in the fibrotic foci were derived from lung epithelial cells in bleomycin‐induced lung injury 4. Uncontrolled lung epithelial cell injury was considered as a key process in the initiation and progression of pulmonary fibrosis by impairing re‐epithelialization and releasing profibrotic cytokines 5, 6. Transforming growth factor (TGF)‐β1 acts as a critical switch in the induction of epithelial‐mesenchymal transition (EMT), which lead to early change in cell morphology, occurring with epithelial cells losing their polarity and acquiring new features of activated fibrogenic phenotype. Such process together with aberrant extracellular matrix (ECM) production will contribute to pulmonary fibrosis 7, 8, 9, 10. Transforming growth factor‐β1 was significantly expressed in epithelial cells of patients with idiopathic pulmonary fibrosis 11 or lung tissues of animals with prolonged pulmonary fibrosis 12.

Connective tissue growth factor (CTGF), a secreted matricellular protein and member of the CTGF/cysteine‐rich 61/nephroblastoma overexpressed family 13, acts in concert with TGF‐β1 to promote and maintain fibrogenesis 14, 15. Connective tissue growth factor was overexpressed in different tissues, such as lung, kidney and liver 16, and plays a critical role in tissue remodelling and fibrosis 17, 18. Re‐distribution of CTGF is mainly derived from activated fibroblasts in pulmonary fibrosis 19. Moreover, CTGF expression was found in lung epithelial cells of tissue samples from patients or animal models with pulmonary fibrosis 20, 21. Recent studies also demonstrated that pulmonary fibrosis could be initiated by an autocrine or paracrine mechanism of CTGF production from activated alveolar epithelial cells 22.

Our previous studies on acute and chronic lung inflammation and airway remodelling suggested that PI3K signalling pathway was involved in the initiation and progression of pulmonary fibrosis by activation of epithelial cells, transdifferentiation of fibrogenic cells and production of ECM 23, 24, 25, 26. The present study aims at investigating potential mechanisms by which TGF‐β1 induces EMT and ECM deposition during the fibrogenesis of lung epithelial cells, and exploring the expression and biological function of CTGF gene and protein in lung epithelial cells. The present study furthermore investigates the involvement of TGF‐β1/PI3K/CTGF activation in EMT and collagen I production induced by TGF‐β1, as well as its role in the biological behaviours of lung epithelial cells.

## Materials and methods

### Cell lines and reagents

Human lung epithelial cell line A549 was obtained from Shanghai Institute for Biological Science. Cells were cultured in RPMI 1640 supplemented with 100 U/ml penicillin, 100 mg/ml streptomycin and 10% heat inactivated foetal bovine serum. Cells were maintained at 37°C in a humidified incubator with 5% carbon dioxide. Human recombinant TGF‐β1 was purchased from R&D Systems China Co. Ltd (Shanghai, China). PI3K inhibitor LY294002 was purchased from Biovision Company (Milpitas, CA, USA). Rabbit monoclonal anti‐ pAkt, Akt, E‐cadherin or Vimentin antibodies were purchased from Cell Signaling Technology Company (Danvers, MA, USA). Rabbit monoclonal anti‐ CTGF and Alpha‐smooth muscle actin (α‐SMA) antibodies and mouse anti‐Collagen I antibody were purchased from Abcam (Hong Kong, China). All genetic manipulation of human cells was approved by the Zhongshan Hospital Research Ethics Committee, Fudan University, China.

### Real‐time PCR

Quantitative RT‐PCR was carried out using real‐time PCR with the SYBR Green reporter. Cell cultures were washed in PBS and RNA was isolated using a guanidinium isothiocyanate/chloroform based technique (Trizol; Invitrogen, San Diego, CA, USA). Optimal density 260 nm was used to determine RNA yield. RNA was subsequently reverse transcribed to cDNA with the SuperScript First‐strand Synthesis System (Invitrogen). Primer concentrations (10 nM) were optimized before use. SYBR Green PCR master kit was used with the appropriate concentrations (10 nM) of forward and reverse primers in a total volume of 20 μl. Quantitative RT‐PCR was carried out using an ABI 7000 PCR instrument (Eppendorf, Hamburg, Germany) with the two‐stage program parameters provided by the manufacturer, as follows: 1 min. at 95°C, and then 40 cycles of 5 sec. at 95°C and 30 sec. at 60°C. Data are shown normalized to GAPDH expression and averaged between three repeated experiments. For data analysis, the raw threshold cycle (CT) value was first normalized to the house keeping gene for each sample to get ∆CT. The normalized ∆CT was then calibrated to control cell samples to get ∆∆CT. Primer sequences were shown in Table S1.

### SDS‐PAGE and Western blot

To measure the expression of EMT and other fibrogenesis related proteins induced by TGF‐β1, cells were cultured at ×10^5^ cells/well and stimulated with vehicle or TGF‐β1 at 1, 5 or 10 ng/ml for 48 hrs. Intracellular protein was extracted by RIPA lysis (Beyotime, Shanghai, China) containing a protease inhibitor cocktail (Selleck, Shanghai, China). Fifty microgram proteins were mixed with an equal volume of 5× SDS sample buffer, boiled for 5 min., and then separated through 10% SDS–PAGE gels. After electrophoresis, proteins were transferred to PVDF membranes by electrophoretic transfer. Membranes were blocked in 5% dry milk for 2 hrs, rinsed and incubated with primary antibodies (diluted at their instructions) in TBS thrice (TBST) at 4°C overnight. Primary antibody was then removed by washing in TBST, and labelled by incubating with 0.1 mg/ml peroxidase‐labelled secondary antibodies (against mouse and rabbit) for 2 hrs. Following three washes in TBST, bands were visualized by ECL (Tanon, Shainghai, China) and exposed to X‐ray film. The band densities were quantified using Image J and the results were expressed as ratio of band density to total actin. And the levels of phosphorylated proteins were assessed with reference to the respective non‐phosphorylated proteins.

### Immunofluorescence staining

Cells at a concentration of 10^4^ cell/ml were seeded onto sterile cover‐slips placed in 24‐well cell culture plates and allowed to grow for 24 hrs. After then, cells were treated with vehicle or TGF‐β1 at 1, 5 or 10 ng/ml for 48 hrs. Cells were fixed with 4% paraformaldehyde for 20 min., washed three times in PBS and permeabilized by 0.1% Triton X‐100 for 20 min. at room temperature. Cells were blocked for 30 min. in PBS containing 10% goat serum and washed thrice. Cells were incubated overnight with primary antibodies: rabbit anti‐E‐cadherin 1:200, Vimentin 1:200, CTGF 1:200, α‐SMA 1:200 and mouse anti‐Collagen I 1:200. Chambers were washed thrice and then incubated with the corresponding secondary antibodies for 1 hr at room temperature. After counterstaining with 4′, 6′‐diamidino‐2‐phenylindole for 5 min., cells were examined for fluorescence under immunofluorescence microscope (Olympus/BX51, Tokyo, Japan).

### RNA interference and transfection

Four different sequences targeted to CTGF mRNA were designed and provided by GenePharma (Shanghai, China). The cells were transferred with sense and antisense strands of shRNAs (Table S1) using Lipofectamine 2000 (Invitrogen) according to manufacturer's protocol. Briefly, 1 μl/well Lipofectamine 2000 was mixed with either 50 pmol/well CTGF or shRNA. The cells were then transfected with shRNA/Lipofectamine complexes in Opti‐MEM (Invitrogen) and incubated for 24 hrs at 37°C. The transfection efficiency was evaluated under fluorescence microscopy and detected by real‐time PCR and Western Blot analysis. Clones with successful transfection were selected for the stable transfection of CTGF and used through experiments.

### Alive measurement of cell bio‐behaviours

The cell bio‐behaviours including total cell number, and cell movement were measured by a Cell‐IQ cell culturing platform (Chip‐Man Technologies, Tampere, Finland), equipped with a phase‐contrast microscope (Nikon CFI Achromat phase contrast objective with ×10 magnification) and a camera (Nikon, Fukasawa, Japan). The equipment was controlled by Imagen software (Chip‐Man Technologies). Images were captured at 5 min. intervals for 72 hrs. Analysis was carried out with a freely distributed Image software (Cell‐IQ Imagen v2.9.5c; McMaster Biophotonics Facility, Hamilton, ON, Canada), using the Manual Tracking plug‐in created by Fabrice Cordelieres (Institut Curie, Orsay, France). Cell‐IQ system with machine vision technology can monitor and record time‐lapse data, and analyse and quantify cell functions and morphological parameters. The movement of each individual cell was measured in the image field by metering the distance of cell movement. Each group contained 6–12 replicate image sites.

### Statistical analysis

Data were represented as mean ± S.E.M. One‐way anova was used for the comparisons of variance among several groups. Dummett's test was used for multiple comparisons. The rates of total cell number were calculated as the following: Rate (%) = (value at each time point value of primary seeding cells)/value of primary seeding cells × 100. Cell movement was calculated as the mean of the distance of every cell moving between two images (5 min. interval). *P*‐values less than 0.05 was considered to be significant.

## Results

**Figure 1:**
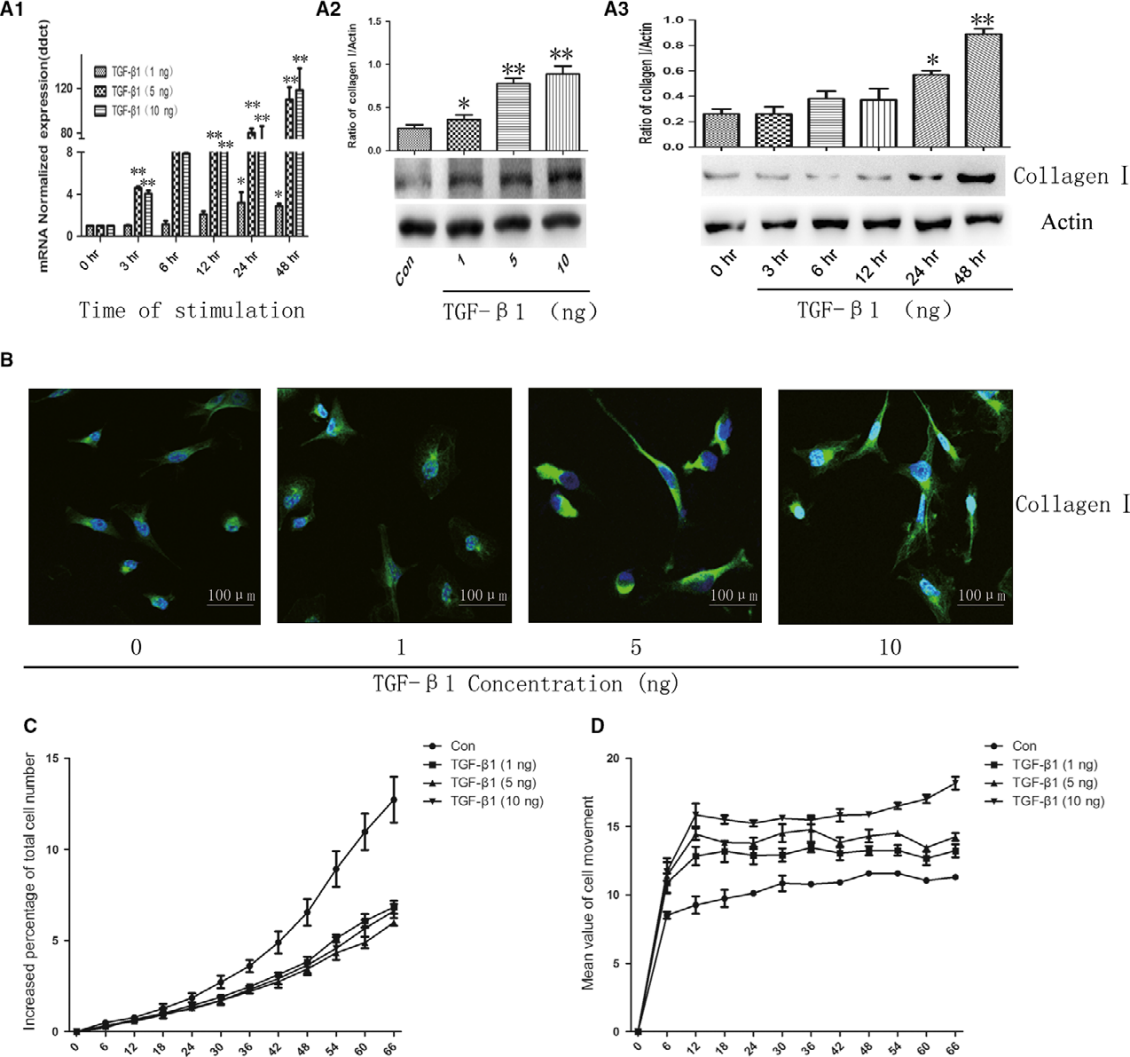
TGF‐β1‐induced collagen I expression. The mRNA (**A1**) and protein (**A2**) of collagen I challenged with vehicle or TGF‐β1 at doses of 1, 5 and 10 ng/ml for an indicated time, respectively. (**A3**) The protein level of collagen I challenged with TGF‐β1 at dose of 5 ng/ml for an indicated time. (**B**) Immunofluorescence staining of collagen I challenged with vehicle or TGF‐β1 at doses of 1, 5 and 10 ng/ml for 48 hrs. Dynamic alterations of the cells proliferation (**C**) and movement (**D**) challenged with vehicle or TGF‐β1 at doses of 1, 5 and 10 ng/ml using Cell‐IQ Alive Image Monitoring System. Data were presented as mean ± S.E.M. of three independent experiments. * and ** stand for *P*‐values less than 0.05 and 0.01 as compared to cells only with vehicle.

To investigate the role of TGF‐β1 in the collagen I deposition. A549 cells were stimulated with different concentrations of TGF‐β1 for an indicated time, the mRNA and protein of collagen I were examined. Quantitative measurement of gene expression by real‐time PCR confirmed a significant increase in collagen I expression in a dose‐ and time‐ dependent manner after TGF‐β1 stimulation (Fig. 1A1). The optimum concentration (5 ng/ml) and time (48 hrs) for TGF‐β1‐induced collagen I expression were chosen for the study and furthermore demonstrated by the results of collagen I protein expression (Fig. 1A2 and A3) and distribution (Fig. 1B). Transforming growth factor‐β1 could decrease cell proliferation (Fig. 1C) and increase cell migration (Fig. 1D), significantly different from those cells treated with vehicle.

**Figure 2:**
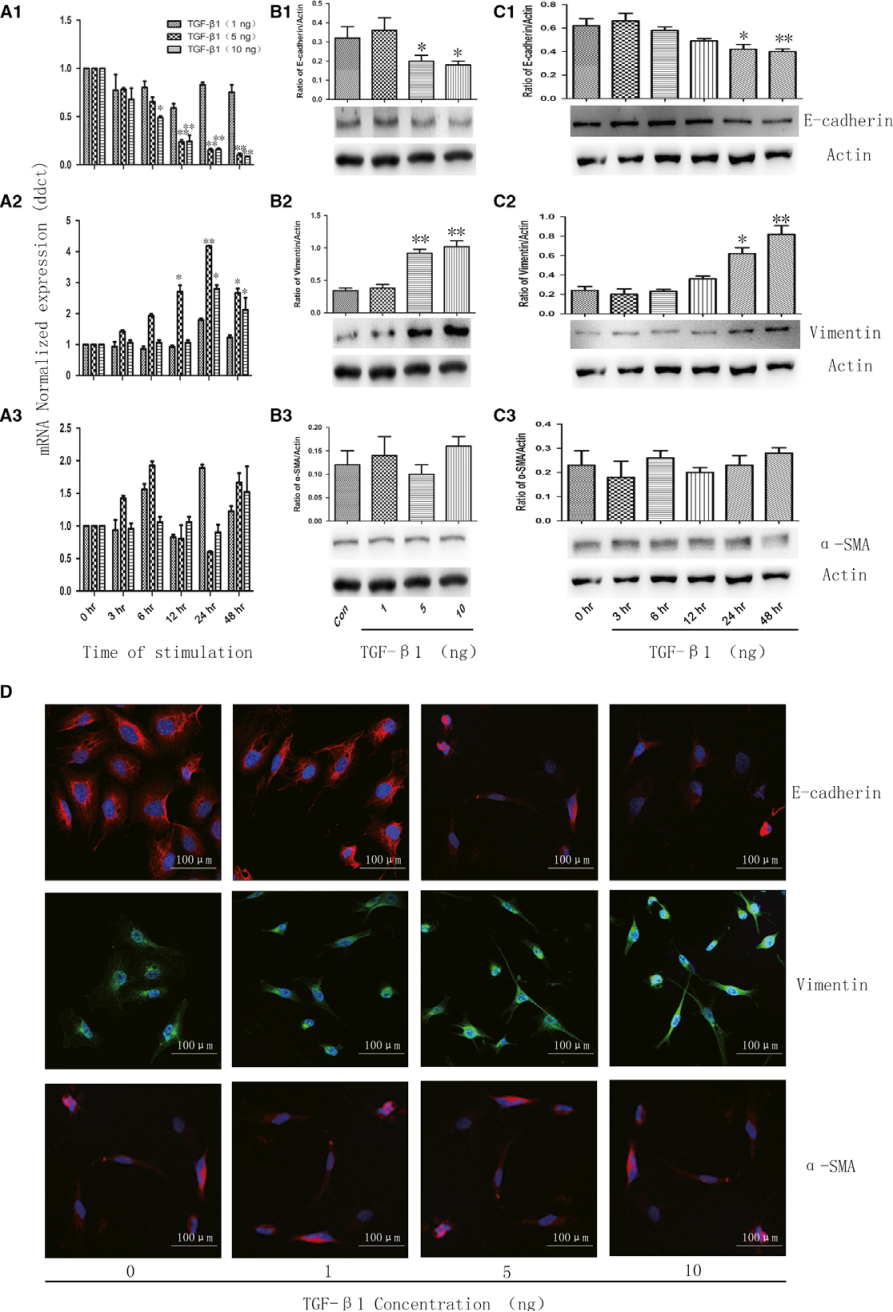
TGF‐β1 induces EMT in A549 cells. The mRNA level of E‐cadherin (**A1**), Vimentin (**A2**) and α‐SMA (**A3**) challenged with vehicle or TGF‐β1 at doses of 1, 5 and 10 ng/ml for an indicated time, respectively. The protein level of E‐cadherin (**B1**), Vimentin (**B2**) and α‐SMA (**B3**) challenged with vehicle or TGF‐β1 at doses of 1, 5 and 10 ng/ml at 48 hrs. The protein level of E‐cadherin (**C1**), Vimentin (**C2**) and α‐SMA (**C3**) challenged with TGF‐β1 at dose of 5 ng/ml at for an indicated time, respectively. (**D**) Immunofluorescence staining of E‐cadherin, Vimentin and α‐SMA challenged with vehicle or TGF‐β1 at doses of 1, 5 and 10 ng/ml for 48 hrs. Data were presented as mean ± S.E.M. of three independent experiments. * and ** stand for *P*‐values less than 0.05 and 0.01, in comparison with vehicle (control) cells.

To characterize the induction of cell EMT phenotype by TGF‐β1, we investigated the epithelial marker E‐cadherin and the mesenchymal marker Vimentin and α‐SMA for an indicated time (Fig. 2). Quantitative real‐time PCR analysis showed a gene expression pattern consistent with EMT including E‐cadherin repression (Fig. 2A1) and the concomitant induction of Vimentin (Fig. 2A2) as compared to control cells. To further study the effects of TGF‐β1 on A549 cells, western blot detected an EMT phenotype protein with results showing that the expression of E‐cadherin significantly decreased with 5 or 10 ng/ml TGF‐β1 after 24 hrs. Inoculation of up to 48 hrs resulted in a further decreased extent of E‐cadherin expression as compared to control cells (Fig. 2B1 and C1), whereas the expression of Vimentin greatly increased after TGF‐β1 stimulation (Fig. 2B2 and C2). In addition, the similar results were demonstrated by immunofluorescence staining (Fig. 2D). The expression of α‐SMA has no statistical difference between different groups (Fig. 2A3, B3 and C3).

**Figure 3:**
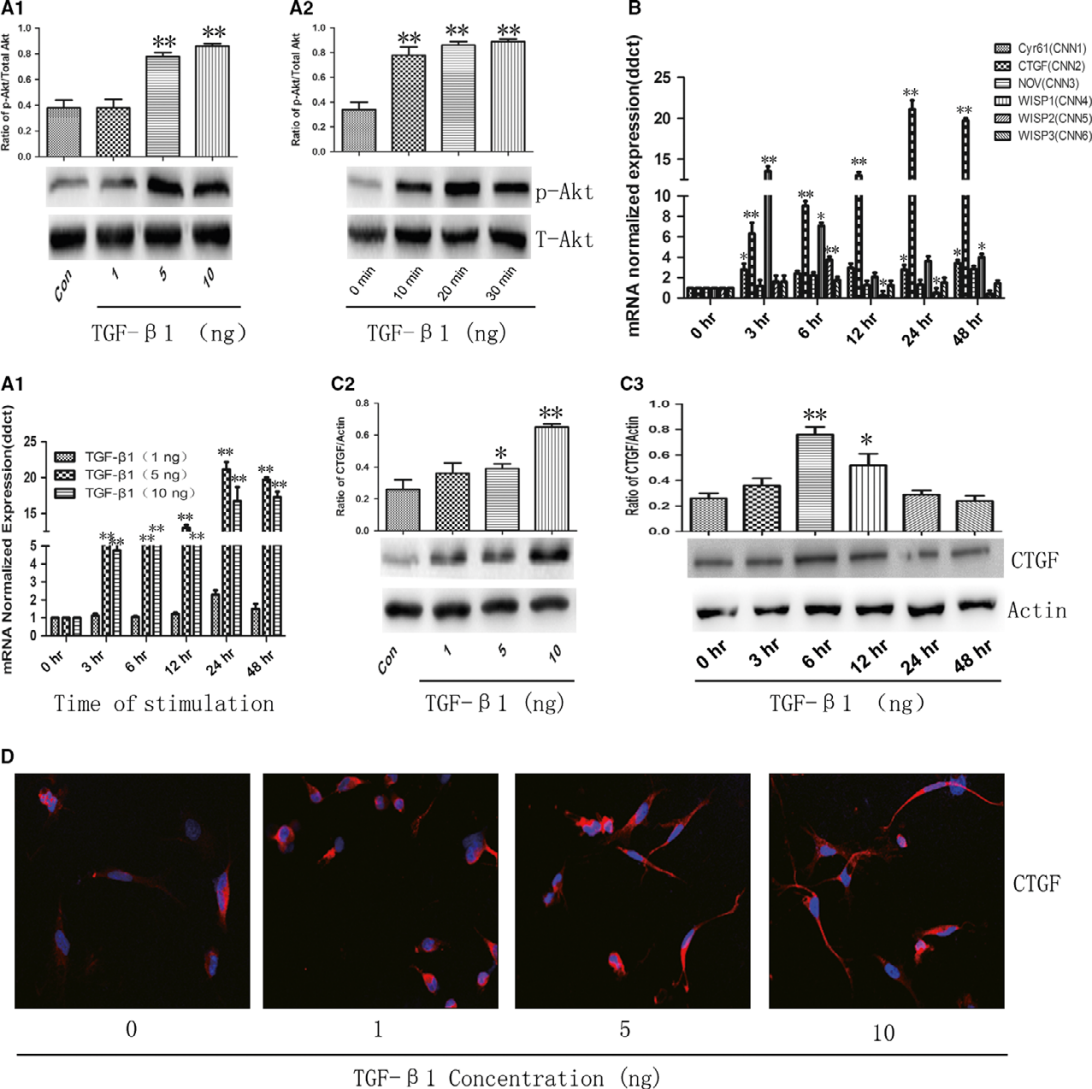
TGF‐β1‐induced PI3K/Akt signalling pathway activation and CTGF production. (**A1**) The level of total Akt and phosphorylated Akt challenged with vehicle or TGF‐β1 at doses of 1, 5 and 10 ng/ml for 10 min. (**A2**) The level of total Akt and phosphorylated Akt challenged with TGF‐β1 at dose of 5 ng/ml for an indicated time, respectively. (**B**) The mRNA level of CNN family (including Cyr61, CTGF, NOV, WISP1, WISP2 and WISP3) challenged with vehicle or TGF‐β1 at dose of 5 ng/ml for an indicated time, respectively. (**C1**) The mRNA level of CTGF challenged with vehicle or TGF‐β1 at doses of 1, 5 and 10 ng/ml for an indicated time, respectively. (**C2**) The protein level of CTGF challenged with vehicle or TGF‐β1 at doses of 1, 5 and 10 ng/ml for 48 hrs. (**C3**) The protein level of CTGF challenged with TGF‐β1 at dose of 5 ng/ml for an indicated time, respectively. (**D**) Immunofluorescence staining of CTGF challenged with vehicle or TGF‐β1 at doses of 1, 5 and 10 ng/ml for 6 hrs. Data were presented as mean ± S.E.M. of three independent experiments. * and ** stand for *P*‐values less than 0.05 and 0.01, in comparison with vehicle (control) cells.

**Figure 4:**
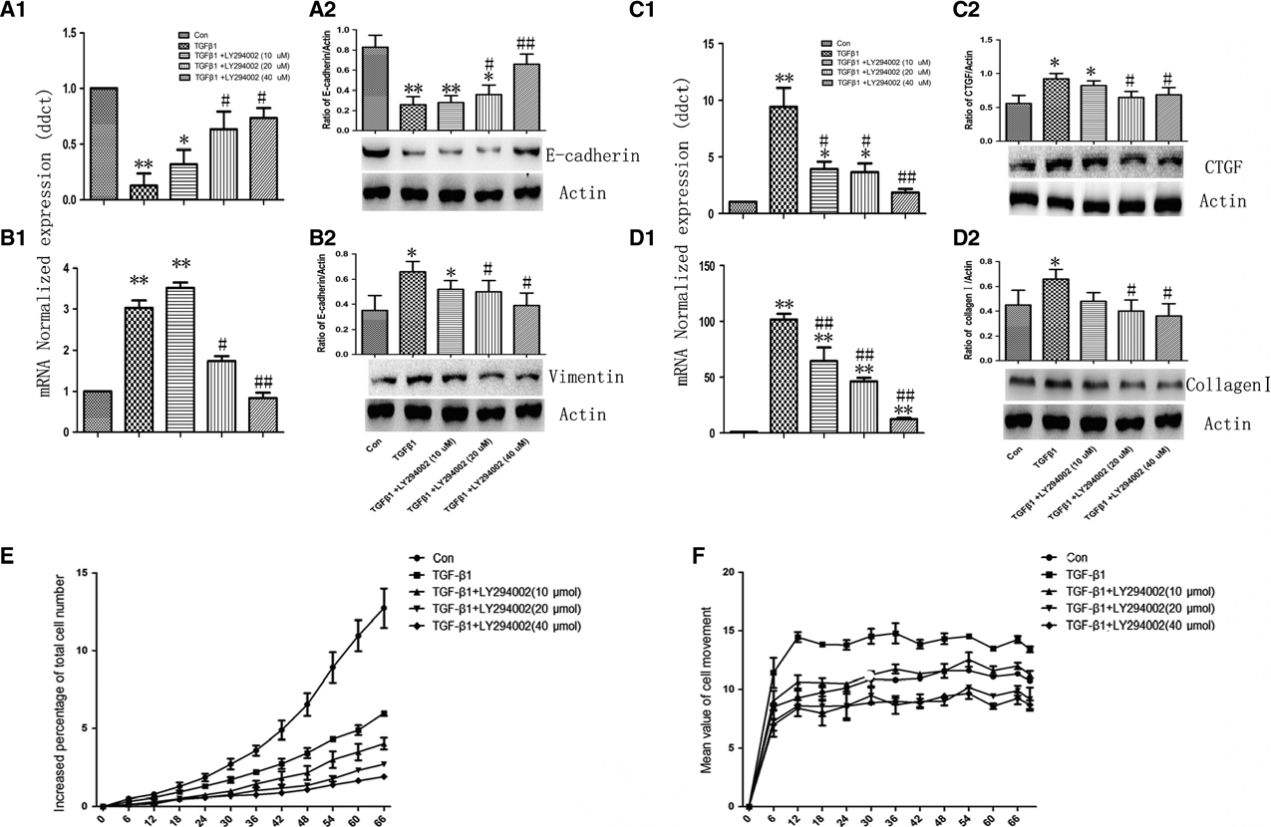
Effects of PI3K inhibitor on TGF‐β1‐induced EMT and collagen I expression. The mRNA and protein level of E‐cadherin (**A1** and **A2**), Vimentin (**B1** and **B2**) and collagen I (**D1** and **D2**) were measured after incubation PI3K inhibitor LY294002 at doses of 10, 20 and 10 μM with TGF‐β1 (5 ng/ml) for 48 hrs. Accordingly, the mRNA and protein level of CTGF (**C1** and **C2**) were measured at 6 hrs. Dynamic alterations of the cells proliferation (**E**) and movement (**F**) using Cell‐IQ Alive Image Monitoring System after incubation PI3K inhibitor LY294002 at doses of 10, 20 and 10 μM with TGF‐β1 (5 ng/ml) for 72 hrs. Data were presented as mean ± S.E.M. of three independent experiments.* and ** stand for *P*‐values less than 0.05 and 0.01 as compared with vehicle (control) cells, and # and ## stand for *P*‐values less than 0.05 and 0.01, as compared to TGF‐β1 (5 ng/ml), respectively.

Our previous study revealed that PI3K signalling pathway played a critical role in the pulmonary fibrosis. To explore whether PI3K signalling pathway was involved in TGF‐β1‐induced ECM deposition, we stimulated A549 cells with 1, 5 or 10 ng/ml TGF‐β1for an indicated time, and found that the phosphorylation of Akt increased at 10 min. and reached at maximum between 20 and 30 min. as compared with the control cells (Fig. 3A1 and A2). The CNN family is an emerging class of inflammation modulators, and the expression of CTGF gene (Fig. 3B and C1) or protein (Fig. 3C2 and C3) increased most in a time‐ or dose‐dependent manner after TGF‐β1 stimulation. Expression and distribution of CTGF protein were up‐regulated from 6 hrs and on after TGF‐β1 stimulation (Fig. 3D). Cells pre‐treated with PI3K inhibitor LY294002 at doses of 10, 20 and 40 μM for 2 hrs were then challenged with vehicle or TGF‐β1 at the concentration of 5 ng/ml for 48 hrs. Treatment with LY294002 significantly reversed TGF‐β1‐induced down‐regulation of E‐cadherin (Fig. 4A1 and A2) and up‐regulation of Vimentin (Fig. 4B1 and B2). The LY294002 also inhibited the expression of CTGF gene and protein (Fig. 4C1 and C2) and collagen I (Fig. 4D1 and D2) induced by TGF‐β1 in a dose‐ or time‐dependent pattern. Additionally, LY294002 could further decrease cell proliferation (Fig. 4E) and migration as compared to cells only treated with TGF‐β1 (Fig. 4F).

**Figure 5:**
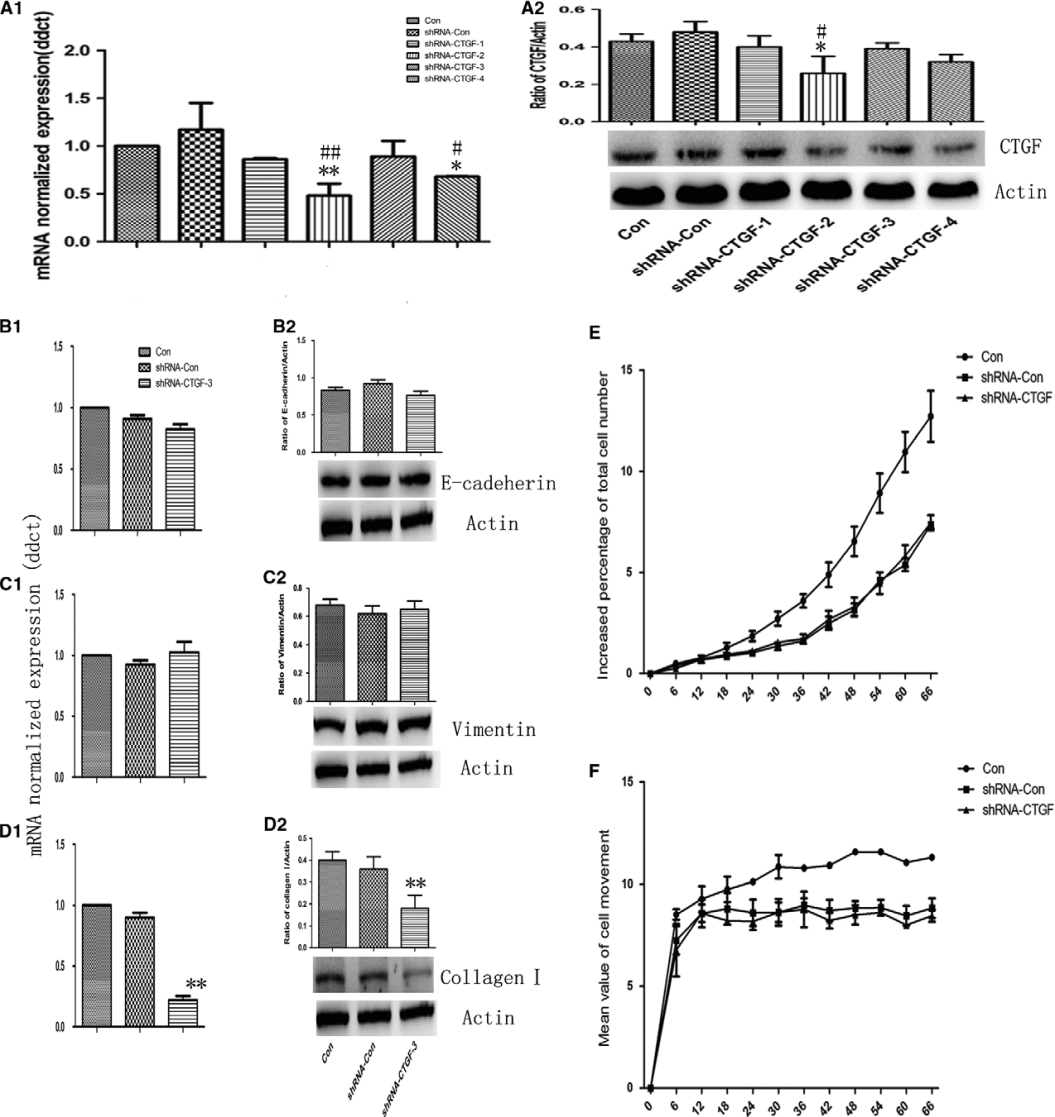
Interference of CTGF expression impaired the basal collagen I synthesis. CTGF in A549 cells was modified by RNA interference, shRNA‐CTGF‐2 was validated for the most efficient interference of CTGF by real time‐PCR (**A1**) and western blot (**A2**). The basal mRNA and protein level of E‐cadherin (**B1** and **B2**), Vimentin (**C1** and **C2**) and Collagen I (**D1** and **D2**) were measured after CTGF interference. Dynamic alterations of the cells proliferation (**E**) and movement (**F**) were measured using Cell‐IQ Alive Image Monitoring System after CTGF interference. Data were presented as mean ± S.E.M. of three independent experiments. * and ** stand for *P*‐values less than 0.05 and 0.01 as compared with vehicle (control) cells, and # and ## stand for *P*‐values less than 0.05 and 0.01, as compared to TGF‐β1 (5 ng/ml), respectively.

**Figure 6:**
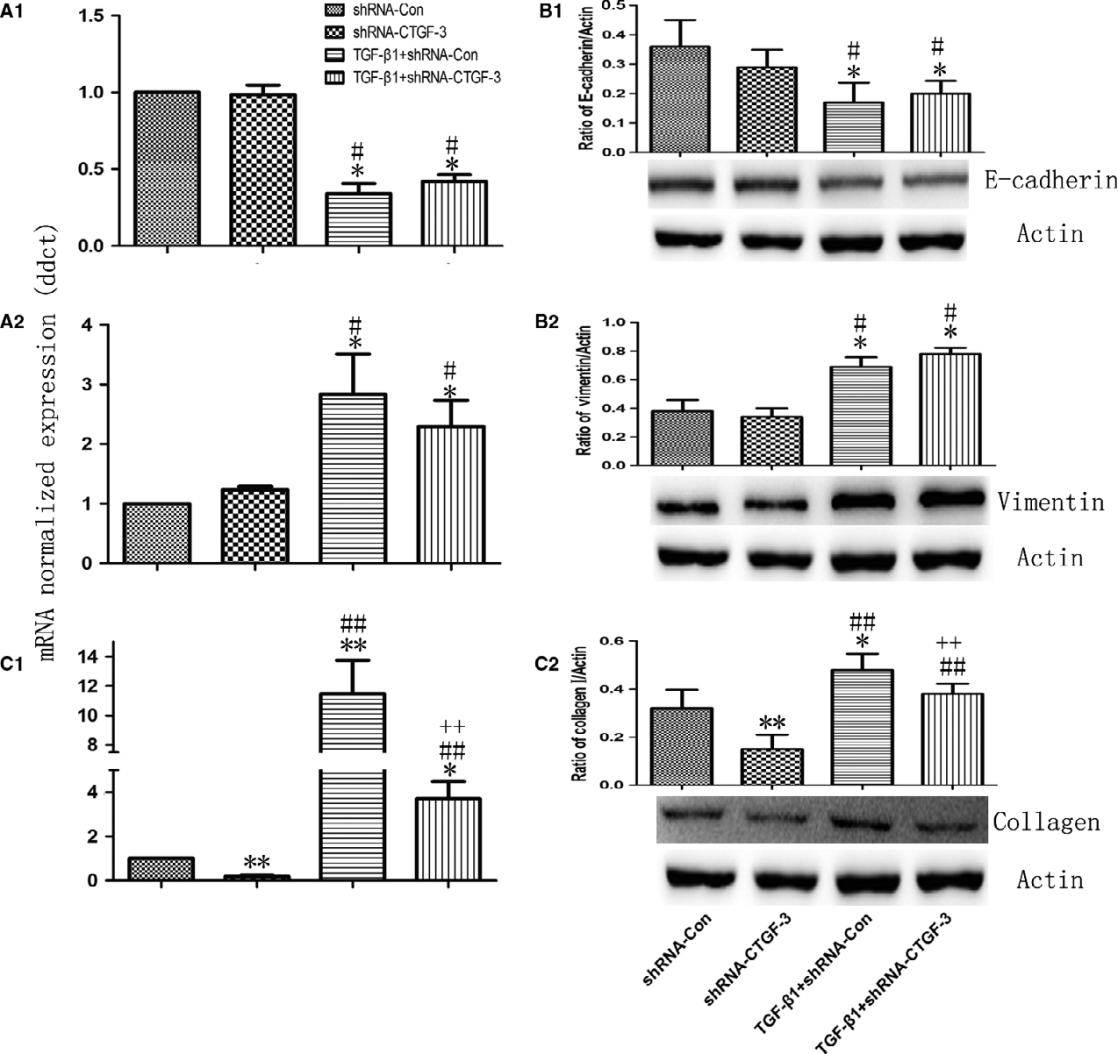
Interference of CTGF expression impaired TGF‐β1‐induced collagen I synthesis. The TGF‐β1‐induced mRNA and protein level of E‐cadherin (**A1** and **B1**), Vimentin (**A2** and **B2**) and collagen I (**C1** and **C2**) were measured after CTGF interference. Data were presented as mean ± S.E.M. of three independent experiments. * and ** stand for *P*‐values less than 0.05 and 0.01 as compared with vehicle (control) cells, and # and ## stand for *P*‐values less than 0.05 and 0.01, as compared with shRNA‐control, + and ++ stand for *P*‐values less than 0.05 and 0.01, as compared with TGF‐β1 and shRNA‐control, respectively.

To further investigate the effect of CTGF on collagen I production and cell behaviours, four pairs of shRNAs were constructed and selected (Fig. 5A1), of which shRNA‐CTGF‐2 showed significantly efficient in the down‐regulation of CTGF (Fig. 5A1 and A2). shRNA‐CTGF‐2 could down‐regulate the expression of collagen I gene (Fig. 5D1) and protein (Fig. 5D2), respectively, as compared to shRNA‐control, while did not influence the expression of E‐cadherin (Fig. 5B1 and B2) or Vimentin (Fig. 5C1 and C2). Both of shRNA‐CTGF‐2 and shRNA‐ control showed slightly inhibitory effects on cell proliferation (Fig. 5E) and migration (Fig. 5F). Transforming growth factor‐β1 failed to increase the expression of collagen I gene and protein in cells^CTGF−/−^ (Fig. 6A3 and B3), while still down‐regulate expression of E‐cadherin (Fig. 6A1 and B1) or up‐regulate expression of Vimentin (Fig. 6A2 and B2).

## Discussion

Pulmonary fibrosis is the end stage of chronic lung diseases, such as lung injury and chronic obstructive pulmonary disease. Fibroblastic foci as the central feature of pulmonary fibrosis is formed by vigorous replication of fibrogenic cells, including interstitial fibroblasts, mesangial cells, bone marrow‐derived fibrocytes or lung epithelial cells 27. The present study provides an experimental evidence that TGF‐β1 could induce lung epithelial cell EMT and ECM synthesis probably through the TGF‐β1/PI3K/CTGF signalling pathway.

The progressive pulmonary fibrotic response is associated with an epithelial‐ dependent fibroblast‐activation process. Repeated injury provokes abnormal activation of lung epithelial cells to secrete various profibrotic cytokines, of which TGF‐β1 was identified to play a primary role in fibrogenesis 28. Lung epithelial cells could contribute to the development of fibroblastic foci through EMC over‐ production after EMT 9. Consistent with previous studies 29, 30, the present study provides further evidence that TGF‐β1 contributes to EMT with changes of characteristic morphology and functional markers. Our results demonstrated that TGF‐β1 could enhance the expression of fibrogenesis‐related proteins, *e.g*. CTGF or collagen I, two important proteins in the pathogenesis of fibrosis. Transforming growth factor‐β1‐stimulated EMT and fibrogenesis of lung epithelial cells could serve as a source of myofibroblast in pulmonary fibrosis 31.

Connective tissue growth factor was first identified as an early gene upregulated in response to growth factors and subsequently determined to be secreted from the active fibroblast, which acts as a functional intermediate between ECM proteins and growth factors 32. The over‐ production of CTGF was found to play a critical role in the progression of various fibroproliferative diseases 18, 33. Rapamycin could regulate CTGF expression in lung fibroblasts and epithelial cells *via* PI3K signalling pathway, resulting in an excessive accumulation of ECM 34, 35. Deletion of CTGF in lung epithelial cells could attenuate bleomycin‐ induced pulmonary fibrosis and collagen I production 22. We found that TGF‐β1‐induced EMT and collagen I synthesis was accompanied with CTGF production. While targeted knockdown of CTGF gene *via* shRNA attenuated both the basal and TGF‐β‐induced collagen I synthesis, rather than the reversal of EMT. Our findings further imply that CTGF serves as a functional intermediate between the TGF‐β1 and ECM proteins. The epithelial‐derived CTGF could activate fibroblasts and further to accelerate the fibrosis process of themselves in an autocrine manner. Inhibition of CTGF production might provide an alternative or adjuvant strategy for TGF‐β1‐induced fibrogenesis.

Transforming growth factor‐β1 could induce EMT and ECM synthesis *via* phosphorylation of Smad 2/3 signalling pathway in human epithelial cells or experimental fibrosis models 36, 37. Additionally, our data and others revealed that there were also some Smad‐ independent signalling pathways involving in TGF‐β1 regulatory mechanism, including the PI3K signalling pathway 38, 39, 40, 41. Our previous studies suggested that the intratracheal delivery of PI3K inhibitors would prevent lipopolysaccharide and pancreatic elastase–induced lung inflammation and remodeling through inhibiting differentiation of lung epithelial cells, activation of myofibroblast, or production of ECM 23, 24. We further explored the potential associative and interactive mechanisms between TGF‐β1‐induced ECM deposition and PI3K signalling pathway in alveolar epithelial cells. The treatment with PI3K inhibitor not only suppressed the CTGF and collagen I synthesis, but also reversed EMT and fibrogenesis of TGF‐β1‐stimulated lung epithelial cells, indicating that TGF‐β1‐Smad dependent and independent signalling pathways work synergistically during fibrosis.

Repeated injury and insufficient regeneration of lung epithelial cells are pathological characteristics of pulmonary fibrosis 42. Chronic inflammation and lung injury could lead to the induction of EMT and the development of pulmonary fibrosis. Our data demonstrated that TGF‐β1/PI3K/CTGF signalling pathway plays an important role in the proliferation and migration of lung epithelial cells. The anti‐fibrotic effect of PI3K inhibitor may be carried out through the inhibition of aberrant proliferation and migration of epithelial or fibrogenic cells.

However, this study is limited by several factors, the alveolar epithelial cell A549 we employed is a transformed, immortalized lung cancer cell line. Although A549 is a well‐established EMT and fibrogenesis cell model, there are still some differences between tumour cells and normal alveolar epithelial cells. Additionally, collagen I is only one of ECM proteins studied in the present study, much more proteins relevant to fibrogenesis need to be furthermore evaluated in future.

**Figure 7:**
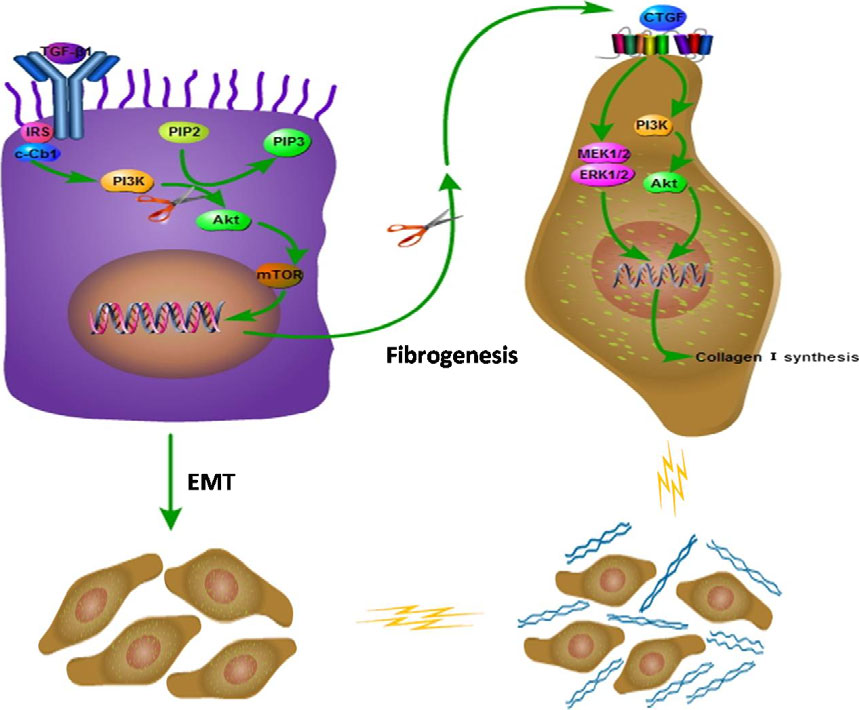
Proposed mechanism of TGF‐β1‐induced fibrogenesis. CTGF, acts as an important downstream effector of TGF‐β1/PI3K signalling pathway, linking TGF‐β1 with ECM production during pulmonary fibrosis. The signal pathway of TGF‐β1/PI3K/CTGF plays an important role in the fibrogenesis of human alveolar epithelial cells, which may be a novel therapeutic approach to prevent and treat pulmonary fibrosis.

In summary, the present study demonstrated that the human lung epithelial cells undergo EMT and ECM deposition after TGF‐β1 stimulation (Fig. 7). Connective tissue growth factor acts as an important downstream effector of TGF‐β1/PI3K signalling pathway to promote ECM production during pulmonary fibrosis. PI3K inhibitor prevented pulmonary fibrosis from lung epithelial cells by reversing EMT and down‐regulation of CTGF and collagen I. Thus, our data indicate that the signalling pathway of TGF‐β1/PI3K/CTGF plays an important role in the fibrogenesis of lung epithelial cells, which may be a novel therapeutic approach for pulmonary fibrosis.

## Disclosure

The authors declare no competing of interests.

## Supporting information


**Table S1** Sequences mentioned in the article.
