# Corrigendum

**DOI:** 10.1111/jcmm.13139

**Published:** 2017-03-10

In Xiangdong Wang 1, page 2185, an error occurred in Figure 2, Panel D, which previously appeared as follows:

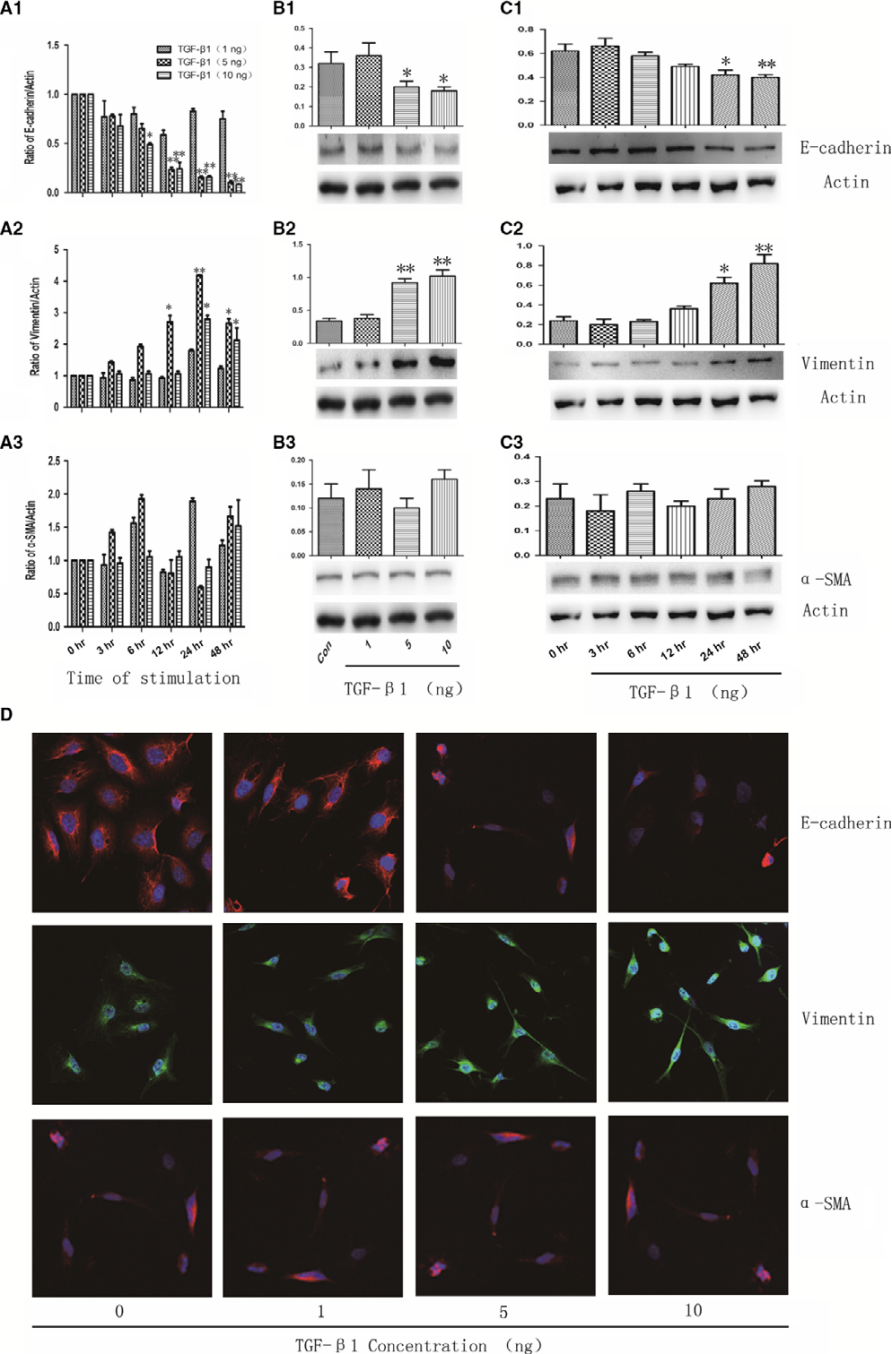

And it should appear as follows:

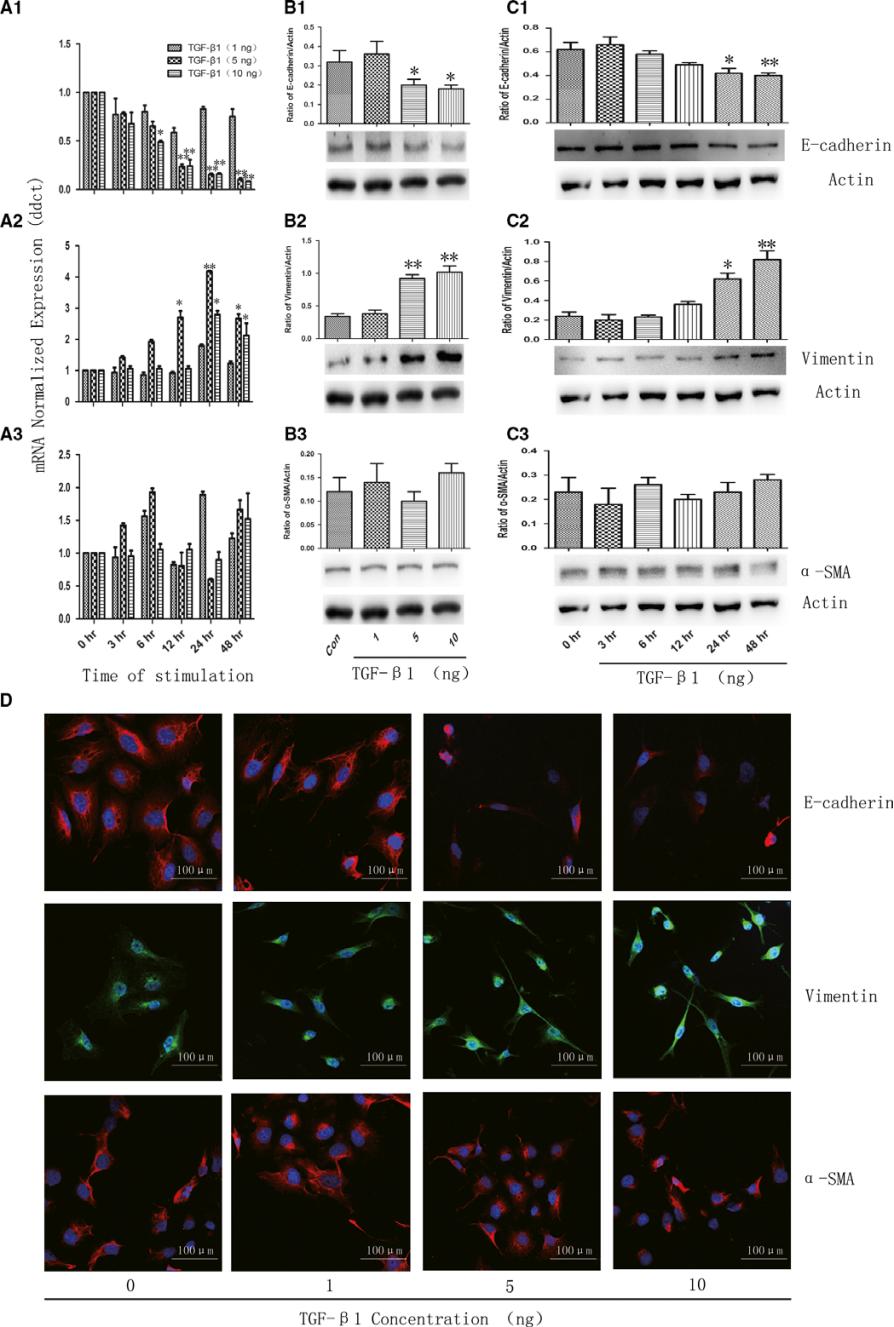

The authors confirm that there are no conflict of interests and wishes to apologize for any misunderstanding or inconvenience caused.
