# Supplementary material for: Regulatory mechanisms of TGF‐β1‐induced fibrogenesis of human alveolar epithelial cells
Source: J Cell Mol Med. 2016 Jul 15;20(11):2183–93. doi: 10.1111/jcmm.12918 (PMC5082411; doi:10.1111/jcmm.12918)
Supplement: Supplementary file 1 — Table S1 Sequences mentioned in the article. [file JCMM-20-2183-s001.doc]

**Additional file 1: Table S1 Sequences mentioned in the article**

| **Name** | **Sequences** | |
| --- | --- | --- |
|  | **Forward primer** | **Reverse primer** |
| **E-cadherin** | 5’- CAGTACAACGACCCAACCCA-3’ | 5’- CACGCTGACCTCTAAGGTGG-3’ |
| **Vimentin** | 5’- AGAACCTGCAGGAGGCAGAAGAAT-3’ | 5’- TTCCATTTCACGCATCTGGCGTTC-3’ |
| **α-SMA** | 5’- CGAGCCGAGAGTAGCAGTTGTAG-3’ | 5’- AGCCATTGTCGCACACGAG-3’ |
| **Collagen Ⅰ** | 5’- TCTAGACATGTTCAGCTTTGTGGAC-3’ | 5’- TCTGTACGCAGGTGATTGGTG-3’ |
| **Cyr61 (CNN1)** | 5’- GCGAGGAGTGGGTCTGTGAC-3’ | 5’CTTGTAAAGGGTTGTATAGGATGC-3’ |
| **CTGF (CNN2)** | 5’- GAGCGGAGAGTCCTTCCAGAG-3’ | 5’- GGCCAAATGTGTCTTCCAGTC-3’ |
| **NOV (CNN3)** | 5’- GCATCTGACCGGCGGTAG-3’ | 5’- CACTGGAATTTGCAGCTTGG-3’ |
| **WISP1 (CNN4)** | 5‘- TGCCCTGGAACACTCCTCTAC-3’ | 5‘- GCACCCACTTCTTGGTTTGAC-3’ |
| **WISP2 (CNN5)** | 5‘- CTGTATCGGGAAGGGGAGAC-3’ | 5‘- AAGAGACAAGGCCAGAAAACTG-3’ |
| **WISP3 (CNN6)** | 5‘- TCCACTCTTCTGCTTGCTGG-3’ | 5‘-GCTGAGGTGCATCTGACACT-3’ |
| **GAPDH** | 5‘- CCACCCATGGCAAATTCCATGGCA-3’ | 5‘TCTACACGGCAGGTCAGGTCCACC-3’ |
| **shRNA-NC** | 5’-UUCUCCGAACGUGUCACGUTT-3’ | 5’-ACGUGACACGUUCGGAGAATT-3’ |
| **shRNA-CTGF-1** | 5’-CCAGACCCAACUAUGAUUATT-3’ | 5’-UAAUCAUAGUUGGGUCUGGTT-3’ |
| **shRNA-CTGF-2** | 5’-CCAAGCCUAUCAAGUUUGATT-3’ | 5’-UCAAACUUGAUAGGCUUGGTT-3’ |
| **shRNA-CTGF-3** | 5’-GCUAAAUUCUGUGGAGUAUTT-3’ | 5’-AUACUCCACAGAAUUUAGCTT-3’ |
| **shRNA-CTGF-4** | 5’-GAAUCGCUGUACUACAGGATT-3’ | 5’-UCCUGUAGUACAGCGAUUCTT-3’ |
